# Variety in Diet

**Published:** 1876-09

**Authors:** Alton Cheswicke


### *VARIETY IN DIET,*

### IN ITS RELATIONS TO HEALTH.

By Alton Cheswicke.

If there is one principle more than another that is firmly established in regard to food, it is that the physical system can not long maintain its functions in a state of vigorous activity—especially in a changeable climate—without more or less frequent change of diet. A prolonged meat diet, for instance, is certain sooner or later, to bring a long list of evils in its train, while, though with fewer attendant evils, perhaps, an eminent gymnast has announced as the result of many years experience, that "increase of strength can not long continue on a diet exclusively vegetable," which is doubtless true in a climate like ours, whatever it might be elsewhere. The system soon becomes cloyed upon any article of food partaken of constantly for any considerable length of time—a sure indication that the wants that this particular kind of food is intended to supply are fully satisfied for the present, and that needs which this can not supply now demand their quota of something else. If the new aliment be not provided, the system breaks out into open rebellion, more or less aggravated according to the extent of the infliction upon its powers of endurance.

It would seem that civilization should afford the most ample means for securing this necessary variety, that in our civilized modes of life we should find this principle of wholesome change and alternation carried out to its fullest perfection. Such, unfortunately, to a very great extent is far from being the case; and it would almost seem as if the savage, when in undisturbed possession of his forests and plains, his hunting-grounds and fishing-streams, had a decided advantage over us in this very respect. For, depending almost entirely upon the spontaneous productions of nature for his subsistence, Nature, like a wise mother as she is, taking into her own hands, as it were, the charge delivered over to her care, gives him only what he needs in each season, and never too much at a time of any one thing. Let us see how she provides for her children.

In the spring, when the system is more or less relaxed by the changing temperature, when consequently appetite—in the adult if not in the growing child—is at its minimum, game is scarce and poor in flesh; so that if the savage eats meat at this season of the year, he must e'en take it as he finds it—in a state of wholesome leanness, and consequently his system is in no danger of becoming over-heated by its use. To compensate for this, however, fish are now coming in from the sea in great numbers and in excellent condition, forming a wholesome and seasonable change, and an agreeable and fitting introduction to summer's more strictly vegetable dietary, and with them, or instead of them, where they are not to be had, are succulent roots which have all winter long been maturing underneath the snows, and are now easily accessible through the softening earth—and such early plants as show themselves ere yet the wintry snows are fairly past; these, with the eggs of the wild fowl and the remnants of last year's store of nuts stolen from the squirrels, and now well mellowed by the frosts, serve to eke out what is perhaps a scanty but eminently wholesome bill of fare; and if at this season the savage keeps rather low in adipose, he at the same time keeps his health, which every one will agree is a fair exchange. For at the season when surfeiting would be attended with the most disastrous results, the means of surfeiting are not at his command, and the time of all the year when his nutriment should be lessened is the very time when there is the least to be had.

Then comes on the glowing summer season. Game is still to a great extent unavailable as food; for the young are still too young to be worth killing, and the parents have not yet begun to accumulate flesh. But game fowl are growing every day more abundant, and though far from fat, are all the more wholesome, for the system neither of man, bird, or brute requires fat at this season of the year. And, indeed, the savage cares little for game of any sort just now, for the milky maize, the ready tribute to his light spring labor, is in its prime, esculent wild vegetables abound, and the plains and hillsides are in all the purple and crimson glory of berries of every sort, the free and generous offering of nature, the fruit of no man's tillage, and the savage revels in a bill of fare that grows every day more varied and abundant, and is most perfectly adapted to his present needs.

By fall, when cool breezes set the blood more vigorously flowing, and the system begins to fortify itself against the coming winter, corn has grown more solid, nutritious, and oily, autumn grains and fruits are ripe and abundant, and game-fowl full-grown and plump, are winging their way southward in countless numbers ; while when winter's keen blasts set a sharp edge on appetite, and all the vital powers are taxed to the utmost by the rigorous demands of the season, and when the system begins to call for a larger supply than it can furnish unaided of oily foods, to keep up a sufficient supply of carbon for atmospheric combustion, then game, fattened by summer's and autumn's abundance, against winter's dearth, are plenty ; their chase affords him the vigorous exercise he requires, and in addition, the first touch of the biting frost sends down showers of ripened nuts from the overladen trees, which serve as winter's provision of bread for man and such animals, as, like him, can not sleep away the wintry months in forgetfulness of hunger. Thus we see that at every season of the year Nature provides the children dependent upon her care with a distinct and varied bill of fare, exactly adapted to the needs of the system, which is thus always supplied with just what it requires, and denies them what would be unnecessary and injurious.

Civilized man should learn a lesson from this provisional arrangement of nature, and pattern after it ; but this is just what he does not do. The resources of civilization enable him not only to multiply, amass, and preserve for future use the distinctive products of every successive season, but also to command the exhaustless abundance of the world at large. They bring to his doors the products of every clime, and enable him to set the season at defiance, and to command the same article of food, the same general bill of fare, if he so wish, the year around, regardless of what nature may have to say about it. Thus, having whatever he may wish for at all times within his reach, convenience, custom, habit, economy, or some other like motive, generally induces him to settle upon a few articles of food which are most prevalent, most easily prepared, cheapest, or most agreeable to his palate, which henceforth become his staples, and are partaken of in equal abundance and with equal freedom all the year round, regardless of the changes of season and climate, and the changing needs of the system. It is all very well for those who, by means of the accessories of civilization, are enabled to surround themselves with a midsummer temperature in the depth of winter, that civilization should place on their tables fruits ripened by tropical suns, and intended to cool the fever of tropical blood, at a time when native fruits are wanting. But, on the other hand, if we are thus enabled to set winter at defiance, and live in a summer temperature the year around, we must also abandon the use of oily, animal, or carbonaceous food, of which the system under this new, artificial regime no longer stands in need. If there is to be no winter in our year, winter's food must be excluded from our table. For, if one habitually surrounds himself with summer temperature in his dwelling, it *is* summer for him no matter what the calendar or the howling winds outside, which come not near him, may say to the contrary; and summer's fare only is permissable. At your peril dare to partake of winter's fare, unless you are willing to face winter's blasts and brave winter's cold!

By way of illustration let us pass in review the principal or staple articles of fare on which the civilized people of these United States—to go no further away from home for example—are wont to regale themselves, and which they seem to regard as indispensable at all times of the year. And first for the great American staple, meat. The English have been called a race of beef and mutton eaters, but it is generally conceded that among the middle classes, especially, which form the bulk of the population, they are fairly outdone in this respect by their American cousins, who are certainly the most carniverous people on the face of the earth. This may have something to do with the indomitable energy which seems to be an inherent feature of the American character; but, on the other hand, unfortunately, there is a dark suspicion that it may also have something—if not as some insist, everything—to do with the long train of ills that American flesh seems pre-eminently heir to. We do not purpose here to denounce the use of animal flesh as food—on the contrary, we hold, individually, that meat is a desirable, and, at certain seasons, an almost indispensable article of diet for *Americans*, especially those residing in the Northern and Northeastern States, whatever it may be for other nations and peoples; but we also claim, on this score, that it is much too good a thing to be abused, as it constantly is.

TO BE CONTINUED.

---

TO CORRESPONDENTS.—We receive many letters, asking us to take up and discuss in our pages a great variety of topics. We are always pleased to thus accommodate our subscribers, but we must ask those who thus desire our assistance to give us their names, so that we may communicate with them by mail if we deem it necessary.

